# Supplementary material for: Chromothripsis during telomere crisis is independent of NHEJ, and consistent with a replicative origin
Source: Genome Res. 2019 May;29(5):737–49. doi: 10.1101/gr.240705.118 (PMC6499312; doi:10.1101/gr.240705.118)
Supplement: Supplemental Material [file supp_gr.240705.118_Supplemental_file_1.zip › contigs/annotated_contigs/DB84/contig.3.DB84_length_349_mean_cov_2.01719197708.docx]

**DB84_length_349_mean_cov_2.01719197708**

```
AGTTAGGATCTGACCAGAAGGGACTCACACTGGTTACATAGGGCATAACTTGAGGGAAAAAAAAAGACAAACACCAACAGCAATTGCAG
 >chr8:36339152-36339412 + E=2e-140
CACACTTAACAATAATAATAATAATAATTTAAAAATACTCAGCACCACAAAATACTTGAAAAATGGCAAGCAAACCCTCTCCTTCTCAG

AAAAATGCAACATCATAGAATTATGTGGTGATAGAAGTAGGTGATAAAACTAGGAAGTCACTGTTTTTTTTTTTTTTTTTT|AGATGTG

GG|TTTGCTCTTGTTGCCCAGGCTTGAGTTC|TATTGCCCG|ATCTCCACTCGCCGCAACCTCCGTCCCCCGGG|CTCTAGTGATTC   >chr6:64160412-64160440 - E=2e-05    >chr2:14021808-14021840 - E=9e-02
```
